# Left Ventricular Pseudoaneurysm Found by CT Scan

**DOI:** 10.2174/1874192400802010026

**Published:** 2008-04-07

**Authors:** Hitoshi Hirose, Iwao Matsunaga, Michael D Strong

**Affiliations:** Department of Cardiothoracic Surgery, Drexel University College of Medicine, Philadelphia, PA. USA

**Keywords:** Left ventricular aneurysm, CT scan, catheterization

## Abstract

A 62-year-old male with a previous coronary artery bypass grafting underwent CT scan for evaluation of left epigastric pain. Findings showed a large left ventricular pseudoaneurysm, which was subsequently confirmed by left ventriculogram. The pseudoaneurysm was successfully repaired surgically.

## INTRODUCTION

The presentation of left ventricular (LV) pseudoaneurysms is often atypical. While the diagnosis is usually established by left ventriculography, the recent improvement in the quality of CT scans has allowed for non-invasive detection. Here we present a patient whose LV aneurysm was found by CT scan.

## CASE PRESENTATION

**Fig. (1):**
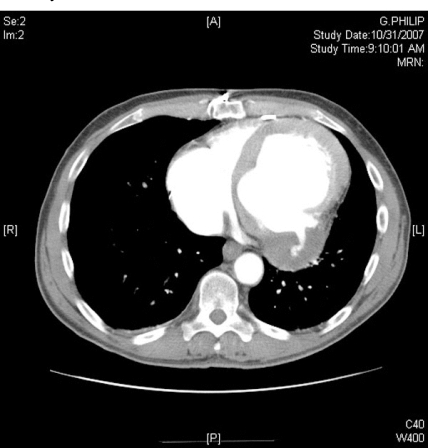
Contrast CT scan demonstrates left ventricular pseudoaneurysm.

**Fig. (2):**
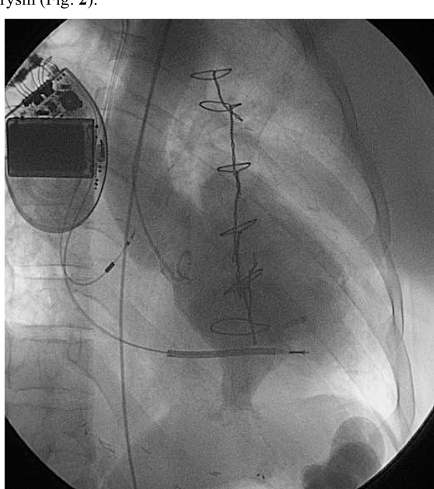
Left ventriculogram demonstrates left ventricular pseudoaneurysm.

A 62-year-old male with a past medical history significant for a coronary artery bypass grafting 10 years prior presented with 3 weeks of persistent left epigastric pain. He denied dyspnea or chest pain. On exam, he was hemodynamically stable, in no apparent distress, and no audible murmur. EKG showed Q wave on the inferior leads suggest ing old myocardial infarct, which was unchanged from a previous EKG performed 3 months prior. A complete series of cardiac enzymes ruled out acute myocardial infarction. Because of the previous coronary bypass surgery and his atypical symptom, the patient underwent cardiac catheterization which showed patent grafts (left internal mammary artery to the left anterior descending artery and saphenous vein graft to the obtuse marginal artery) and intact right coronary system. Left ventriculography was not performed at that time. Transthoracic echocardiography showed severely decreased left ventricular function. Because of persistent pain in the left epigastric area to the left flank, a contrast CT scan was obtained to role out intraabdominal pathology. The 16 slice spiral CT scan with intravenous contrast demonstrated a large LV pseudoaneurysm (Fig. **1**). There was no other intraabdominal lesion was observed. Left ventriculogram was subsequently performed and confirmed the LV pseudoaneurysm (Fig. **2**).

**Fig. (3):**
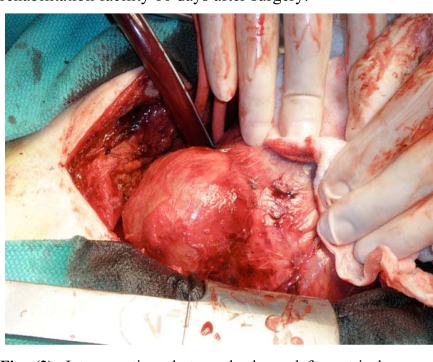
Intraoperative photograph shows left ventricular pseudoaneurysm located in the posterolateral wall of the left ventricle. The pseudoaneurysm was filled with organized thrombus.

The patient was then scheduled for resection of the LV pseudoaneurysm. After dissection of the anterior wall of the heart, further dissection of the lateral and posterior wall of the heart was performed under cardiopulmonary bypass because of dense adhesion. The LV pseudoaneurysm was located in the posterolateral wall of the LV (Fig. **3**), adherent to the posterior pericardium. After complete immobilization of the LV, the pseudoaneurysm was opened under aortic cross clamp. The wall of the pseudoaneurysm was thin and filled with clots. The neck of the pseudoaneurysm was identified and closed with a Gore-Tex patch. The ventriculotomy was then closed with multiple buttressed sutures. The postoperative course was uneventful and he was discharged to a rehabilitation facility 10 days after surgery.

## DISCUSSION

Pseudoaneurysms of the LV are rare complications of myocardial infarction. The pathogenesis results from microperforation and contained rupture of the LV occurring at the time of the acute myocardial infarction. The overlying adherent pericardium over the clots prevent from free rupture, then a pseudoaneurysm will develop. No specific symptoms of the LV pseudoaneurysm have been described. While patients may be asymptomatic, they can also present with symptoms of heart failure or atypical chest pain [1].

The diagnosis of LV pseudoaneurysm is usually made by echocardiography and/or left ventriculogram, but establishing the diagnosis can be difficult. One study showed that 10% of LV aneurysms were diagnosed incidentally [1]. Left ventriculography is the gold standard for LV pseudoaneurysm diagnosis and provides a definitive diagnosis in more than 85% of patients [1]. Transesophageal echocardiography can be an alternative method to diagnose LV pseudoaneurysm with a diagnostic accuracy of 75%. [1, 2].In contrast, it is often difficult to diagnose LV pseudoaneurysm with transthoracic echocardiography. In such a setting, cardiac MRI could be helpful to distinguish myocardium form thrombus [2].

Previously, the usefulness of CT scan for detecting LV aneurysm has been limited because of artifact created by cardiac motion. However, in the case presented, advances in the CT scan technology allowed us to make a correct diagnosis of LV pseudoaneurysm. Our patient underwent contrasted CT scan to rule our intraabdominal lesion due to persistent epigastric pain. The CT scan interestingly disclosed LV pseudoaneursym without intraabdominal lesion. Epigastric pain of this case was most likely from the compression effect due to the LV pseudoaneurysm. The contrasted 16 slice successfully provided a precise image of the pseudoaneurysm, which was subsequently confirmed with left ventriculography. Contrast CT should be considered an alternative, non-invasive modality for diagnosing LV pseudoaneurysm.

## References

[1] Frances C, Romeo A, Grady D (1998). Left ventricular pseudoaneurysm. J Am Coll Cardiol.

[2] Cho MN, Mehta SK, Matulevicius S, Weinstein D, Wait MA, McGuire DK (2006). Differentiating true versus pseudo left ventricular aneurysm.A case report and review of diagnostic strategies. Cardiol Rev.

